# Production of a Complementary Food: Influence of Cowpea Soaking Time on the Nutritional, Antinutritional, and Antioxidant Properties of the Cassava-Cowpea-Orange-Fleshed Potato Blends

**DOI:** 10.1155/2020/8873341

**Published:** 2020-10-28

**Authors:** Abiola Folakemi Olaniran, Clinton Emeka Okonkwo, Omorefosa Osarenkhoe Osemwegie, Yetunde Mary Iranloye, Yemisi Tokunbo Afolabi, Omokolade Oluwaseyi Alejolowo, Charles Obiora Nwonuma, Toluwanimi Esther Badejo

**Affiliations:** ^1^Department of Food Science and Nutrition, College of Agricultural Sciences, Landmark University, P.M.B. 1001 Omu-Aran, Kwara State, Nigeria; ^2^Department of Agricultural and Biosystems Engineering, College of Engineering, Landmark University, P.M.B. 1001 Omu-Aran, Kwara State, Nigeria; ^3^Department of Microbiology, College of Pure and Applied Sciences, Landmark University, P.M.B. 1001, Omu Aran, Nigeria; ^4^Department of Industrial Chemistry Programme, Department of Physical Sciences, College of Pure and Applied Sciences, Landmark University, P.M.B. 1001, Omu Aran, Nigeria; ^5^Department of Biochemistry, College of Pure and Applied Sciences, Landmark University, P.M.B. 1001, Omu Aran, Nigeria

## Abstract

Soaking and incorporation of legumes for fortification are essential to a complementary food production process. Cassava, orange-fleshed potato, and cowpeas are sustainably cheap, locally available, and underutilized for food biofortification. This study investigated the effect of cowpea soaking time (3, 6, and 9 h) on different composition ratios of cassava, cowpea, and orange-fleshed sweet potato (CCP) blends (50 : 40 : 10 (EC), 50 : 30 : 20 (FC), 50 : 20 : 30 (GC), and 50 : 50 : 0 (HC)). Each blend was assayed for pH, antinutrient, antioxidant, and proximate contents. Results obtained showed that the CCP blends were significantly influenced by the length of cowpea soaking. Moisture and fiber content decreased significantly (*P* ≤ 0.05) with increased steeping time (3 to 9 h) for the cassava-cowpea-OFSP blends. The blends were significantly different (*P* ≤ 0.05) in terms of their protein, fiber, fat, ash, and carbohydrate contents. The moisture content of the EC blend was significantly different from only FC and HC blends, respectively. Six (6) hours of soaking showed no significant difference in the nutritional composition of the flour samples compared with 9 hours. The soaking length optimizes the health and nutrient-promoting factors in the various blend samples while also reaffirming cowpeas as a viable biofortification option for use in complementary food production.

## 1. Introduction

Soaking generally involves the submergence of seeds in water at room temperature. This may be followed by either dehulling or cooking depending on the expected end product. It has become accepted as a viable process amenable to several technological applications. It is more recommended by scientists particularly for bean processing for centuries [1]. Soaking is primarily a household technology revered for the treatment and processing of complementary foods. It was reported to preserve the health benefits and enhances the nutritive values of complementary food products [2]. Koffi-Nevry et al. [3] reported that soaking induces antinutritional losses through diffusion. Thus, soaking represents a technological process for the removal of soluble compounds and may attract application as an alternative to decrease the antinutritional contents of foods [4]. Cowpea grains contain 25% protein in the diet, and this has made it acceptable for use in different traditional African dishes that involved grain or seed soaking [5]. Legume seed cotyledon is the prime photosynthetic producer and reservoir of diverse organic nutrients; hence, it is rich in starch and protein. This has led to the exploration of many methods in the production of complementary foods purposefully to increase their energy gains from consuming them [6].

Cassava is grown all seasons, cheap and a stable source of carbohydrates. It is of importance in many household menus during periods of food scarcity. In addition, its propagation is a major source of income for the middle-class population and its industrial relevance cannot be overemphasized [7]. Nigeria is currently the largest producer of cassava in Africa with over 50 million tons per annum which accounts for 61% of the continent's production in Africa, and 58% in the world [8]. Similarly, potato ranks third on the list of most essential food crops globally and exceeded the consumption index of other crops by being the highest consumed with more than a billion individuals worldwide [9]. Orange-fleshed sweet potato is a rich source of provitamin A [10]. Currently, reports abound on the positive effects of potato on the wellbeing of people and this has facilitated the integration of biofortified orange-fleshed sweet potato (OFSP) into the diets of some African countries like Kenya, Uganda, Nigeria, and Mozambique as reported by Neela and Fanta, [11]. Low et al. [12] also reported the protection of preschool age children from night blindness when exposed to diets with 125 g of boiled OFSP. This suggests potato as a potential option in improving children's daily vitamins' requirement in foods. Food crops like cassava, potato, and some legumes, especially cowpea, are cheap and apparently underexplored for commercial health food production [13].

Complementary foods are vital in meeting the infants' nutritional requirements and may suffice for mothers with breast milk feeding challenges or whose break milk failed to provide the optimum nutrient requirements of infants [14]. Common complementary foods for infant's gruels are usually prepared by the fermentation of cereals such as maize, sorghum, and millet [15]. The prices of imported foods used for instant preparation of gruels are frequently high and above the financial capacity of many middle- to low-income households' mothers [16]. Furthermore, the addition of OFSP to infant food formula and household diets can reduce the risk of vitamin A deficiency. This further expands the health food options of less privileged families beyond green vegetable and fruit sources for vitamin A [17]. The cassava-based weaning food formulas have been reported using a blend of cassava, cocoyam, white potato, cowpea, etc. as materials [18–21]. Conversely, very little reported cases from Nigeria exist on the influence of the cowpea soaking time on the nutrient and organoleptic statuses of complementary foods. This study therefore is aimed at investigating the effect of varying soaking time of cowpea grains on the antioxidant, proximate, and nutritional indices of different blends of cassava-based weaning food formulas.

## 2. Materials and Methods

### 2.1. Raw Material Collection

Orange-fleshed sweet potato (OFSP) (*Ipomoae batatas)* and cassava tubers (*Manihot esculenta Crantz*) were harvested from Landmark University Teaching and Research Farm, Omu-Aran. Brown variety of cowpea (*Vigna unguiculata*) grains was obtained from a reputable local food store outlet, Kwara State. The proximate analysis of the raw materials used for the food formulation is shown in Table 1.

### 2.2. Soaking of Cowpea Grains

The cowpea grains were sorted to remove broken grains and stones. Cleaned and unblemished cowpea grains (6 kg) were packed into 3 bags of 2 kg each for processing. Each pack was steeped in 6 liters of tap water in transparent plastic buckets at room temperature (25 ± 2°C) for 3, 6, and 9 hours, respectively. The grains were later manually dehulled as described by Maseta et al. [22] with modification by varying the soaking time. The dehulled grains of each batch were labeled C3 (soaked for 3 hours), C6 (soaked for 6 hours), and C9 (soaked for 9 hours), respectively. Labeled samples were used for further processing.

### 2.3. Production of Precooked Cowpea Flour

Five hundred grams each of previously labeled dehulled cowpea grains were steamed in 1 liter of boiling water for 15 minutes. The steamed cowpea grains in each batch were drained in a strainer and allowed to cool. The samples were dried in an oven (Memmert UF75) for 12 hours at 60°C. Subsequently, the dried precooked cowpea samples were milled into a fine flour (2 mm mesh size) with the aid of a laboratory hammer mill (armfield miller) and packaged and stored as described by Rodríguez-Sandoval et al. [23] and Olaniran et al. [18] for further use.

### 2.4. Production of Fermented-Frozen-Precooked Cassava Flour

Freshly harvested cassava tubers were weighed, washed, and peeled. The peeled cassava tubers were diced into cubes, rinsed, drained, and reweighed. Three kilograms of the diced cassava was soaked in 9 liters of water for 48 hours in a closed plastic container. The fermented cassava was later drained and steamed in boiling water for 15 minutes before draining. These precooked cassava samples were allowed to cool at room temperature and placed in the freezer (-20°C) for 24 hours. The frozen samples were sliced into 3 mm thin sheets and thawed and dried for 12 hours at 60°C on stainless steel trays in a Memmert UF75 oven. They were allowed to cool and milled into fine flours using the hammer mill and weighed prior to being packaged.

### 2.5. Orange-Fleshed Potato Flour Production

Five [5] kilograms of freshly harvested orange-flesh potato (OFSP) tubers were washed under running tapwater and peeled. The peeled potato was diced into thin sheets (3 cm each) and dried for 12 hours at 60°C. The dried potato sheets were milled into flours with the hammer mill, and the resultant flour was weighed, packaged, and stored at room temperature (26 ± 2°C) in accordance with the method of Alawode et al. [24].

### 2.6. Formulation of Cassava-Cowpea-Orange-Fleshed Sweet Potato (OFSP) Blend

The formulation ratio used for the cassava-cowpea-orange-fleshed sweet potato (OFSP) flour blend and labels were 50 : 40 : 10 (EC), 50 : 30 : 20 (FC), 50 : 20 : 30 (GC), and 50 : 50 : 0 (HC), respectively. The blends were properly stirred using a handheld mixer for 5 minutes. The control is the flour of the unsoaked cowpea. The formulated samples were weighed into plastic bags in triplicates [18] for further analysis.

### 2.7. Determination of Total Titratable Acidity

The TTA (total titratable acidity) of all the cassava-cowpea- orange-fleshed sweet potato (OFSP) blends was determined. 1 g of each sample was reconstituted in 10 ml of distilled water and vigorously shaken. Three drops of phenolphthalein were added as the indicator before the mixture was titrated against 0.1 M NaOH. Each ml of the 0.1 M NaOH used was equal to 0.09 of lactic acid. The titer value was taken by using the final and initial readings in triplicates to calculate the mean values [25].

### 2.8. pH Determination

The pH meter (Hi 2550, Hanna Instruments) was used to determine the pH of the formulated cassava-cowpea-orange-fleshed sweet potato (OFSP) blends in triplicates. 1 g of flour was reconstituted in 10 ml of distilled water and shaken vigorously [25].

### 2.9. Determination of Proximate Composition and Mineral Content

Analysis of the formulated cassava-cowpea-orange-fleshed sweet potato (OFSP) blend for crude fat, protein, ash composition, crude fiber, starch content and total reducing sugar, calcium, and phosphorus contents were done in triplicate as described by AOAC [25].

### 2.10. Quantitative Determination of Terpenoids and Alkaloids in the Formulated Complementary Food

Two (2) g of the sample was placed in 250 ml beaker and soaked with 50 ml 95% ethanol for 24 hours at room temperature. The mixture was filtered, and the residue was extracted with petroleum ether (60–80°C) and concentrated to dryness. Total terpene content was expressed in terms of mg/g [26]. Alkaloids were determined using the procedure of Henry (1973) as described by Uddin et al. [27]. The addition of 30 ml of 80% alcohol to 3 g of the sample was carried out to make a smooth paste then made up to 100 ml. One gram (1 g) of magnesium oxide was digested for 90 minutes in a boiling water bath under a reflux air condenser shaken at interval. The mixture was filtered through a funnel. The residue was placed in a flask and redigested for 30 minutes with 50 ml alcohol. The alcohol was evaporated and hot water was added to replace the alcohol lost. With all alcohol removed, two to three drops of 10% HCl was added. The resulting solution was transferred into a 150 ml volumetric flask. Five (5) ml of zinc acetate solution and 5 ml of potassium ferrocyanide solution were added, carefully mixed to give a homogenous solution and allowed to stand for 5minutes. Filtrated through a dry filter paper, 10 ml of the filtrate was pippeted into a separating funnel for extraction of alkaloids by vigorously shaking with 150 ml portions of chloroform with 30 ml added at a 60-second interval for 5 times. The resultant residue was dissolved in hot water and transferred into a Kjeldahl flask. Sucrose (0.2 g), 10 ml of concentrated H_2_SO_4_, and selenium (0.02 g) were added and digested into a colorless solution. The percentage of NH_3_ was determined by Kjeldahl's distillation method. Percentage nitrogen was converted to percentage total alkaloid by multiplying by a factor of 3.26.

### 2.11. Determination of the Total Phenolic Content of the Formulated Flour

Total phenolic content (TPC) of the methanolic extracts obtained from the formulated flours was determined using the Folin-Ciocalteu reagent, as described by Olaniran and Abiose [28] with minor modifications. The extracts were dissolved in ethanol and were added to test tubes, as described: 0.12 ml of the extracts' solutions (15 ml), 0.3 ml of the Folin-Ciocalteu reagent (0.2 M), and 2.4 ml of sodium carbonate 5% solution (40 g l^−1^), to obtain a final concentration of 15 mg l^−1^ and then added to the test tubes. The mixture was shaken and heated at 40°C in a water bath for 20 min. The tubes were then cooled rapidly, and the developed color was read at 767 nm in a UV-Vis spectrophotometer (model Mutispec-1501, Shimadzu, Japan). The concentration of phenolic compounds was estimated using a calibration curve traced with gallic acid (GA) in ethanol as a polyphenol reference, in triplicate. The results were expressed as mg of GA equivalents/g of extract (mg·GAE·g^−1^).

### 2.12. Radical Scavenging Activity of the *α*,*α*-Diphenyl-*β*picrylhydrazyl Radical (RSA-DPPH)

The antioxidant capacity of flour samples was measured in terms of their radical scavenging ability (RSA), using the DPPH method as described by Olaniran and Abiose [29]. The extract (150 mg l^−1^) and DPPH (0.04 mg l^−1^) were dissolved in ethanol. Each ethanol extract (0.30 ml, 15 mg l^−1^) was mixed with 2.7 ml of DPPH solution (32 *μ*g ml^−1^) to give a final sample concentration of approximately 15 mg l^−1^. The mixture was homogenized and stored in the dark before analysis. Spectroscopic evaluation of the antioxidant activity was performed at 767 nm in a UV-Vis spectrophotometer (model Mutispec-1501, Shimadzu, Japan). The percentage of the DPPH radical scavenging activity (RSA%DPPH) of each sample was calculated as follows: %RSA = (1–AC/AD) × 100, where AC is the absorbance of the solution when the extract was added at a particular concentration after 30 min, and AD is the absorbance of the DPPH solution; all the analyses were performed in triplicate. The IC_50_ (half-maximal inhibitory concentration) or I% (percentage of inhibition) was calculated graphically using a calibration curve in the linear range by plotting the extract concentration against the corresponding scavenging.

### 2.13. Evaluation of Vitamin A and Lycopene of Formulated Blends

The formulated blends (1 g) were weighed into 125 ml flasks wrapped with aluminum foil to exclude light. A 50 ml mixture of n-hexane-acetone-ethanol (2 : 1 : 1; *v*/*v*/*v*) was added to solubilize the carotenoids' content. Each flask was stoppered and agitated continuously for 30 min on a magnetic stirrer plate until lycopene was completely extracted. This was confirmed in the colorless pulp fibers. Agitation was continued for another 2 min after adding 10 ml of water. The solution was then allowed to separate using a separation funnel into distinct polar (35 ml) and nonpolar (25 ml) layers containing lycopene. The hexane solution containing lycopene was filtered through a 0.22 *μ*m filter paper before measuring the absorbance using a spectrophotometer at 472 nm [30]. The conversion of absorbance into lycopene concentration was based on its specific extinction coefficient (E1cm1%) of 3450 in hexane using the Beer Lambert equation. For estimation of vitamin A, the solution was measured in the spectrophotometer at 450 nm.

### 2.14. Statistical Analysis

All the experiments were carried in triplicate; the mean and standard deviation were calculated using Microsoft Excel 2016. Analysis of variance (ANOVA) and Duncan multiple range tests were carried using IBM SPSS statistics 22 to test the significance of soaking time at *P* ≤ 0.05.

## 3. Results and Discussion

### 3.1. Proximate and Nutritional Composition of the Cassava-Cowpea-OFSP Blends

The influence of steeping time (3 to 9 h) on the formulated blends EC (50 : 40 : 10), FC (50 : 30 : 20), GC (50 : 20 : 30), and HC (50 : 50 : 0) is presented in Table 2. An inverse relationship was observed between the moisture contents of the different blends and the soaking time of cowpea grains. It is suffice to say that moisture decreased significantly (*P* ≤ 0.05) with increased soaking time, with FC blend having the highest moisture content of 9.73% at cowpea soaking time of 3 h, while the lowest (9.06%) was observed for EC at 9 h cowpea soaking. This may be due to the rate of water uptake and the nature and structure of the cowpea seeds. The result agreed with previous studies during the soaking of cowpea, lima bean, and Bambara groundnuts for production of complementary food, respectively [21, 31, 32]. The moisture content of the formulated complementary foods obtained in this current study was below 10% which is significant for the prevention of microbial growth and the extension of the product shelf life [18, 33]. According to Laryea et al. [34], the low moisture content from this current study is an indication of high dry matter and energy (calorific) contents.

The protein content of EC and FC blends oscillated across soaking time. Increase in cowpea soaking time resulted in protein increase of GC. This might be attributed to the quantity of steeped cowpea flour in the blends. The highest protein value (29.34%) was recorded for EC (6 h of steeping), while the lowest protein content (20.83%) was in GC (3 h of steeping).

The observed increase in protein content during the study might be due to modifications from the activities of microorganisms on fiber, lipid, starch, and ash contents of steeped cowpea [35]. Ojokoh et al. [36] reported the positive effect of steeping length on the nutrient composition of cowpea flour, particularly protein. The variations in protein content of EC, FC, and HC observed in this current study were due to the length of steeping coupled with possible leaching of their nitrogenous constituent [37]. Fernandes et al. [2] observed that bean processing reduces protein content but increases its digestibility.

There were significant differences in crude fiber contents of the formulated sample as the steeping time increased. The highest fiber content (1.92%) was observed in the EC blend with cowpea steeped for 3 h during processing and FC blend with cowpea steeped for 9 h was the lowest (1.03%). Soaking has been reported to reduce the soluble dietary fiber in dry beans comprising of carbohydrate which are soluble in water but cannot be digested in the human stomach,thus, leaving the insoluble dietary fiber which can be broken down, forming prebiotic compounds, providing energy for probiotic organisms, and improving the maintenance of healthy digestive system [38].

Crude fat steadily increased for EC from 1.23 to 1.33% but was observed to be slightly unstable for FC, GC, and HC. The fat content for FC and GC initially showed a decrease (3 to 6 h) from 1.81 to 1.76 and 1.81 to 1.71%, respectively. These values increased afterward, whereas HC crude fat increased from 1.83 at 3 h steeping to 2.81% at 6 h and slightly decreased afterward to 2.05 at 9 h soaking. The highest fat content of 2.81 ± 0.02% was noted for HC sample at 6 h of steeping and the lowest (1.26 ± 0.01%) was observed for EC at 3 h steeping. This observation is similar to that of Neela and Fanta [11] in a flour composited with OFSP. Soaking was reported to result in an increase in the lipid content of soybean flour. This may be the result of the leaching of the soluble components which causes an increase in the concentration of lipids in the flour [1]. The fat content of the complementary food samples in this study is also in agreement with WHO standards [39]. Fat is important in the diets of infants and young children because it provides essential fatty acids, facilitates absorption of fat-soluble vitamins, and improves dietary energy density and sensory qualities according to Pan American Health Organization (PAHO) and World Health Organization [40].

Ash value of the formulated foods also increased significantly till the 6^th^ hour for EC. The ash content for FC, GC, and HC initially increased from 3 to 6 h and later declined afterward. The highest ash content (7.83%) was observed for HC at 6 h of steeping, while GC had the lowest value of 5.22% at 3 h of steeping. A decrease in ash content was reported by Adenuga [41] and Amagloh et al. [42] from their respective studies involving sweet potato-based complementary foods. This current study showed a relatively higher ash content in all the formulated samples than previously reported by Adisetu et al. [43] who also worked with OFSP complementary foods. The inconsistency in the ash content across OFSP complimentary foods studies may be attributed to differences in maceration and dehulling time which have strong implications for the minerals' solubility [3].

The carbohydrate content of the blends showed a similar trend across steeping time. It decreased from 3 to 6 h and increased afterward at 9 h. The highest carbohydrate value (61.21 ± 0.076%) was noted for the GC blend at 3 h of steeping while EC had the lowest (51.68 ± 0.113%) at 6 h of steeping. Cowpea steeping for 3 to 6 h in the production of the sample formulation improved carbohydrate values which declined at 9 h due to its rapid utilization by microorganisms for growth and development by microorganisms during their exponential growth phase. This is in agreement with the study that suggested the generation of more amylose-based compounds during the soaking period and reported similar patterns with heat application which facilitated leaching and solubilization [44]. The blends were significantly different in terms of their protein, fiber, fat, ash, and carbohydrate contents. However, EC formulation moisture content was significantly different from that of FC and HC probably due to the composite ratio of the different sample formulations.

### 3.2. Biochemical Composition of the Cassava-Cowpea-Orange-Fleshed Sweet Potato (OFSP) Blend

The effect of steeping time on some mineral composition of the formulated blends is represented in Table 3. The sugar content of the EC blend decreased significantly with steeping time. It was however notably inconsistent for FC, GC, and HC. FC and GC blends showed decrease in the sugar content from 3 h to 6 h soaking time but increased afterward at 9 h, while HC blend showed increase in the sugar content at 3 to 6 h steeping time to decline afterward. The highest value of sugar (4.82 mg/100 g) was observed for HC at 6 h of steeping, while GC had the lowest value of 3.55 mg/100 g at GC at 6 h. Soaking length of dry seeds used in the production of formulated foods has been reported to markedly reduce the total soluble sugar and starch contents [45]. This is due to the partial solubilization of the total sugar level in legume grains thus removed through discarding of soaking water [46]. The calcium content showed a steady decrease with increase steeping for the EC and FC blends to later increase at 9 h and fluctuated for GC (increased from 3 to 6 h and decreased afterward) and HC (decreased from 3 to 6 h of steeping and increased afterward), respectively. It was observed that the percentage values of calcium and phosphorous increased cowpea processing. A similar observation was reported by Felix et al. [47] while studying the effect of soaking on *Adenanthera pavonina* seeds.

A similar trend was noted for the phosphorus content in all the sampled blends across the steeping time with a steady increase from 3 to 6 h and a decrease afterward. The HC blend had the highest phosphorus value (4.65 mg/100 g) at 3 h of steeping while the lowest value (3.07 mg/100 g) was observed for the GC blend at 3 h of steeping. There was an observed decrease in calcium with increase in soaking time. Reduction after soaking in calcium assayed may be attributed to leaching into the soaking medium [48]. The results obtained for phosphorus and calcium across the different sample formulations were not statistically significant (*P* ≤ 0.05). Soaking has been reported to improve bioavailability of minerals by increasing its solubility in legumes thus its nutritional quality [49]. The percentage level of the calcium and phosphorus in the formulated food conforms to the WHO standard despite the relatively higher values noted for phosphorus [50].

### 3.3. Effect of the Soaking on the pH and Total Titratable Acidity of the Cassava-Cowpea-Orange-Fleshed Sweet Potato (OFSP) Blend

The pH increased significantly for EC and GC, decreased for HC, and fluctuated for FC. At 3 and 9 h of steeping, there was no significant difference. The highest pH (6.97) was at the HC blend (3 h of steeping), while the lowest pH (6.41) was for the FC blend (9 h of steeping). The total titratable acidity (TTA) of the blend increased significantly for GC across the steeping time but was observed to be inconsistent for EC, FC, and HC. The TTA decreased from 3 to 6 h of steeping for EC and FC blends and increased afterward, whereas there was an increase from 3 to 6 h in TTA values for HC followed by a decrease from 6 to 9 h steeping. The highest TTA value (1.06) was for EC (9 h of steeping), and the lowest was for EC (6 h of steeping). The blends were significantly different in their calcium and pH content but not statistically different for sugar, fat, ash, and carbohydrate contents; phosphorus; and total titratable acid. An increase in the total titratable acidity with a decrease in the pH was recorded during the steeping process. The weak acidic values obtained from the blends may be due to the direct influence of lactic acid bacteria during the steeping of the cowpea grains [51]. The interactive potential of the different composites used in the sampled formulations might have also influenced the TTA.

### 3.4. Antioxidant Properties and Antinutrient Composition of the Cassava-Cowpea-Orange-Fleshed Sweet Potato (OFSP) Blends

The results of the analysis on the antioxidant property of the blends are shown in Table 4. Total phenolic content (TPC) significantly increased with steeping time for EC, GC, and HC but fluctuates across the steeping levels for FC. The GC blend had the highest total phenolic content value (826.98 mg GAE/g) at GC 9 h of steeping, and the lowest (269.84 mg GAE/g) at 3 h of steeping for an HC formulated sample. There was no marked increase or decrease in total antioxidant activities (TAA) of the blends across soaking levels. Generally, the formulated blends showed better total antioxidant activities (1.04-0.70 IC_50_) compared to the control sample (1.94 IC_50_) with the highest activity recorded in GC (0.70 IC_50_). Lesser values of IC_50_ show higher antioxidant activities [28]. The protective effect of beans and some legumes against certain chronic diseases has been associated with the presence of phenolic compounds [52, 53]. Fernandes et al. [2] reported an inverse correlation between the total phenolic content and antioxidant activity of beans and some leguminous seeds. This validated the total antioxidant activity results obtained in the current study.

Terpenoid showed inconsistent values that are not significantly different (*P* > 0.05) across the steeping levels in the formulated blends except in FC that had a steady decrease in terpenoid content across steeping time. The highest terpenoid value of 10.81 mg/100 g was observed for GC (6 h of steeping), while the FC and HC had the lowest value (10.41 mg/100 g) at 9 h and 3 h, respectively. Also, the alkaloid content of the formulated samples decreased progressively across steeping time consequently suggesting a strong correlation with increasing soaking length. The highest alkaloid value was observed for the GC blend composed of cowpea soaked for 3 h while HC had the lowest at 9 h of steeping. This could be explained by the fact that terpenoid and alkaloids are lost during processing due to their high sensitivity to oxidation and leaching into water-soluble media during storage. The results obtained from this study suggest that the different formulated samples have both health-promoting and antinutritional factors. Processing techniques such as soaking, cooking, germination, and fermentation have a reducing effect on the levels of antinutritional factors. These may include phytates, phenols, tannins, and enzyme inhibitors. The reduction is facilitated by the release of exogenous and endogenous enzymes such as phytase enzymes during processing. Consequently, processing increases the bioavailability of minerals such as iron and zinc;, improves protein digestibility, and attracted more attention to sorghum as a source of food. The observed reduction of the antinutrient content in flour formulated from cowpea steeped for 9 hours may be due to processing across steeping time [33]. Abeshu and Kefale [54] reported that tubers contain a significant percentage of alkaloids and terpenoid; this might suggest the level of variation values in the blend. Terpenoids have been reported as an important antioxidant with anti-inflammatory and antimicrobial potential [27].

Lycopene increased in FC blend between 3 and 6 h of steeping and GC (6 to 9 h) but fluctuated for EC and HC. There was no significant difference in the lycopene content of the sampled blends between 3 and 6 h of steeping. The highest lycopene content (0.29 mg/l) was noted in GC (9 h of steeping), while the lowest value (0.02 mg/l) was for HC (3 and 9 h of steeping). Soaking time had no significant effect on the vitamin A content of the sampled blends. The GC blend (0.02 mg/l) had the highest vitamin A content while EC, FC, and HC blends had the lowest vitamin A content. The blends were not significantly different in their vitamin A content but significantly different in their total phenolic content, lycopene, total antioxidant activities, alkaloid, and terpenoid contents. Studies have shown that intakes of foods rich in lycopene protect humans against diseases associated with oxidative stress [55] and have no reported case of side effect or genotoxicity [56].

### 3.5. Relationship between the Proximate and Antioxidant Property of the Blends

The correlation between the proximate composition and antioxidant property from the study is shown in Table 5. Moisture content had a significant positive relationship (*P* ≤ 0.05) with alkaloid (0.324^∗^). Protein correlated significantly with terpenoid (0.39^∗^), alkaloid (-0.35^∗^), radical scavenging activity (-0.39^∗^), and lycopene (-0.38^∗^). Fat correlated with terpenoid (0.51^∗∗^) and radical scavenging activity (-0.4^∗^). Ash had a strong significant relationship (*P* ≤ 0.01) with total phenolic content (-0.89^∗∗^), terpenoid (-0.74^∗∗^), radical scavenging activity (0.91^∗∗^), lycopene (-0.57^∗∗^), and vitamin A (-0.63^∗∗^). The carbohydrate correlated significantly with alkaloid (0.38^∗^), lycopene (0.6^∗∗^), and vitamin A (0.54^∗∗^). The positive correlation between ash and radical scavenging activity showed that an increase in ash will increase the radical scavenging activity value of the blend, whereas the negative correlation between ash, total phenolic, terpenoid, lycopene, and vitamin A showed that as the ash content of the blends increase, the total phenolic content, terpenoid, lycopene, and vitamin A would decrease; this supports the findings shown in Tables 2 and 4. It is, therefore, very important to complement roots and tubers with cowpea with soaking as pretreatment to improve their health, mineral, and nutritional values in the processes for developing complementary food.

## 4. Conclusion

The study has established that soaking of cowpea as a processing technique during production of cassava-cowpea-orange-fleshed potato flour optimized the essential nutrients in complementary food for a healthy and nutritional quality. Corroborating cowpeas are a viable option for use in complementary food production. The findings from the study also showed that the formulation of cassava-cowpea-orange fleshed potato flour blends were significantly influenced by the duration of cowpea soaking. Soaking of cowpea for six [6] hours is recommended as optimum in cassava-cowpea-orange-fleshed potato flour blend production as there were no significant differences in the nutritional composition of the flour compared to blends formulated from 9 hours of soaking.

## Figures and Tables

**Table 1:** Proximate composition of the raw materials used for the food formulation.

Proximate (%)	Cowpea	Orange-fleshed sweet potato	Cassava
Moisture	9.23 ± 0.025	78.20 ± 0.002	65.58 ± 0.022
Protein	19.78 ± 0.010	2.42 ± 0.001	4.01 ± 0.015
Fiber	1.54 ± 0.010	1.04 ± 0.005	5.31 ± 0.000
Fat	0.83 ± 0.010	0.77 ± 0.031	0.54 ± 0.010
Ash	9.56 ± 0.010	1.52 ± 0.006	2.49 ± 0.005
Carbohydrate	59.06 ± 0.015	16.05 ± 0.001	22.07 ± 0.011

*n* = 3.

**Table 2:** Proximate composition of the cassava-cowpea-orange fleshed sweet potato (OFSP) blend.

Sample code	Steeping time (h)	Moisture (% wet basis)	Protein (%)	Fiber (%)	Fat (%)	Ash (%)	Carbohydrate (%)
EC	3	9.36 ± 0.071^c^	28.29 ± 0.266^b^	1.92 ± 0.010^b^	1.26 ± 0.010^b^	6.57 ± 0.020^a^	52.60 ± 0.295^b^
	6	9.20 ± 0.006^b^	29.34 ± 0.095^d^	1.78 ± 0.006^c^	1.28 ± 0.006^d^	6.71 ± 0.010^c^	51.68 ± 0.113^a^
	9	9.06 ± 0.010^a^	28.44 ± 0.012^c^	1.56 ± 0.010^a^	1.33 ± 0.012^c^	6.73 ± 0.012^b^	52.92 ± 0.042^b^
FC	3	9.73 ± 0.006^c^	24.64 ± 0.012^b^	1.81 ± 0.010^b^	1.81 ± 0.010^b^	6.21 ± 0.010^a^	56.81 ± 0.006^b^
	6	9.60 ± 0.015^b^	26.76 ± 0.015^d^	1.44 ± 0.270^c^	1.76 ± 0.010^d^	6.56 ± 0.085^c^	53.89 ± 0.371^a^
	9	9.50 ± 0.026^a^	24.86 ± 0.586^c^	1.03 ± 0.015^a^	1.83 ± 0.015^c^	6.03 ± 0.015^b^	56.74 ± 0.600^b^
GC	3	9.32 ± 0.030^c^	20.83 ± 0.021^b^	1.75 ± 0.010^b^	1.81 ± 0.015^b^	5.22 ± 0.042^a^	61.07 ± 0.076^b^
	6	9.22 ± 0.040^b^	22.92 ± 0.015^d^	1.61 ± 0.020^c^	1.70 ± 0.015^d^	6.80 ± 0.0150^c^	57.75 ± 0.015^a^
	9	9.11 ± 0.025^a^	23.26 ± 0.085^c^	1.13 ± 0.010^a^	1.82 ± 0.020^c^	6.13 ± 0.012^b^	58.57 ± 0.080^b^
HC	3	9.36 ± 0.015^c^	25.03 ± 0.010^b^	1.34 ± 0.010^b^	1.83 ± 0.021^b^	7.57 ± 0.012^a^	54.87 ± 0.040^b^
	6	9.21 ± 0.015^b^	24.75 ± 0.068^d^	1.27 ± 0.010^c^	2.81 ± 0.010^d^	7.83 ± 0.020^c^	54.13 ± 0.036^a^
	9	9.23 ± 0.015^a^	23.74 ± 0.051^c^	1.26 ± 0.015^a^	2.05 ± 0.025^c^	7.34 ± 0.015^b^	56.38 ± 0.142^b^

*n* = 3. EC = 50 : 40 : 10, FC = 50 : 30 : 20, GC = 50 : 20 : 30, and HC = 50 : 50 : 0 of Cassava-cowpea-OFSP blend. Similar letter denotes not significant, while different letters denotes significant.

**Table 3:** Biochemical composition of the cassava-cowpea-orange-fleshed sweet potato (OFSP) blend.

Sample code	Steeping time (h)	Sugars (mg/100 g)	Calcium (mg/100 g)	Phosphorus (mg/100 g)	pH	TTA
Control	0	3.8 ± 0.015^a^	9.30 ± 0.01^a^	2.4 ± 0.01^a^	5.81 ± 0.01^a^	0.06 ± 0.01^a^
EC	3	4.6 ± 0.012^c^	15.49 ± 0.055^c^	3.86 ± 0.015^c^	6.52 ± 0.02^b^	0.99 ± 0.012^c^
	6	4.58 ± 0.012^b^	13.78 ± 0.026^d^	4.15 ± 0.015^d^	6.61 ± 0.015^c^	0.5 ± 0.015^b^
	9	4.5 ± 0.01^d^	13.14 ± 0.061^b^	3.76 ± 0.057^b^	6.76 ± 0.081^b^	1.06 ± 0.056^d^
FC	3	4.46 ± 0.015^c^	11.93 ± 0.021^c^	3.55 ± 0.01^c^	6.56 ± 0.01^b^	0.91 ± 0.01^c^
	6	4.44 ± 0.01^b^	11.9 ± 0.015^d^	3.7 ± 0.01^d^	6.78 ± 0.01^c^	0.81 ± 0.015^b^
	9	4.61 ± 0.01^d^	10.80 ± 0.05^b^	3.31 ± 0.01^b^	6.41 ± 0.01^b^	1.05 ± 0.015^d^
GC	3	4.52 ± 0.021^c^	9.73 ± 0.015^c^	3.07 ± 0.115^c^	6.42 ± 0.015^b^	0.74 ± 0.006^c^
	6	3.55 ± 0.01^b^	13.17 ± 0.061^d^	4.12 ± 0.025^d^	6.53 ± 0.01^c^	0.8 ± 0.015^b^
	9	4.43 ± 0.015^d^	9.78 ± 0.02^b^	3.38 ± 0.025^b^	6.53 ± 0.021^b^	0.85 ± 0.006^d^
HC	3	4.33 ± 0.015^c^	15.55 ± 0.01^c^	4.65 ± 0.092^c^	6.97 ± 0.01^b^	0.82 ± 0.021^c^
	6	4.82 ± 0.015^b^	15.07 ± 0.11^d^	4.63 ± 0.025^d^	6.87 ± 0.02^c^	0.96 ± 0.01^b^
	9	4.54 ± 0.005^d^	15.47 ± 0.015^b^	4.27 ± 0.015^b^	6.75 ± 0.006^b^	0.78 ± 0.02^d^

Control: cowpea at no steeping time; TTA: total titratable acid. EC = 50 : 40 : 10, FC = 50 : 30 : 20, GC = 50 : 20 : 30, and HC = 50 : 50 : 0 of Cassava-cowpea-OFSP blend. Similar letter denotes not significant, while different letters denotes significant.

**Table 4:** Antioxidant properties of the cassava-cowpea-orange-fleshed sweet potato (OFSP) blend.

Sample code	Steeping time (h)	TPC (mg GAE/g)	Terpenoid (mg/100 g)	Alkaloid (mg/100 g)	TAA (IC_50_)	Lycopene (mg/l)	Vitamin A (mg/l)
Control	0	87.26 ± 1.415^a^	8.18 ± 0.035^a^	4.45 ± 0.076^c^	1.94 ± 0.02^b^	0.005 ± 0.0001^a^	0.003 ± 0.006^a^
EC	3	674.05 ± 0.961^b^	10.16 ± 0.021^b^	4.53 ± 0.011^d^	0.91 ± 0.01^a^	0.03 ± 0.001^b^	0.01 ± 0.0002^b^
	6	675.13 ± 0.108^c^	10.13 ± 0.01^c^	3.6 ± 0.015^b^	0.88 ± 0.015^a^	0.04 ± 0.001^b^	0.01 ± 0.0001^b^
	9	678.28 ± 0.065^d^	10.19 ± 0.02^b^	2.12 ± 0.021^a^	0.88 ± 0.015^a^	0.03 ± 0.001^c^	0.01 ± 0.0001^b^
FC	3	813.07 ± 0.05^b^	10.13 ± 0.02^b^	5.07 ± 0.115^d^	0.74 ± 0.01^a^	0.07 ± 0.004^b^	0.01 ± 0.0002^b^
	6	813.36 ± 0.169^c^	10.13 ± 0.01^c^	3.07 ± 0.115^b^	0.74 ± 0.015^a^	0.07 ± 0.004^b^	0.01 ± 0.005^b^
	9	815.23 ± 0.025^d^	10.04 ± 0.015^b^	2.26 ± 0.015^a^	0.76 ± 0.015^a^	0.07 ± 0.002^c^	0.01 ± 0.0001^b^
GC	3	820.65 ± 0.006^b^	10.32 ± 0.026^b^	5.9 ± 0.015^d^	0.72 ± 0.015^a^	0.2 ± 0.002^b^	0.02 ± 0.0001^b^
	6	823.46 ± 1.01^c^	10.81 ± 0.015^c^	4.71 ± 0.01^b^	0.71 ± 0.01^a^	0.2 ± 0.002^b^	0.02 ± 0.0001^b^
	9	826.98 ± 0.03^d^	10.23 ± 0.015^b^	2.52 ± 0.012^a^	0.70 ± 0.015^a^	0.29 ± 0.001^c^	0.02 ± 0.0001^b^
HC	3	269.84 ± 0.772^b^	10.04 ± 0.021^b^	4.17 ± 0.026^d^	1.03 ± 0.02^a^	0.02 ± 0.016^b^	0.01 ± 0.0002^b^
	6	280.74 ± 0.28^c^	10.16 ± 0.031^c^	3.05 ± 0.035^b^	1.04 ± 0.026^a^	0.03 ± 0.002^b^	0.01 ± 0.0002^b^
	9	302 ± 0.881^d^	10.16 ± 0.045^b^	1.82 ± 0.015^a^	1.04 ± 0.025^a^	0.02 ± 0.001^c^	0.01 ± 0.0002^b^

Control: cowpea at no steeping time; TPC: total phenolic content,: TAA: total antioxidant activities. EC = 50 : 40 : 10, FC = 50 : 30 : 20, GC = 50 : 20 : 30, and HC = 50 : 50 : 0 of the Cassava-cowpea-OFSP blend. Similar letter denotes not significant, while different letters denote significant across column.

**Table 5:** Correlation between proximate composition and antioxidant properties of the formulated blend.

	Moisture	Protein	Fiber	Fat	Ash	CHO	TPC	Terpenoid	Alkaloid	TAA	Lycopene	Vitamin A
Moisture	1											
Protein	0.02	1										
Fiber	-0.45^∗∗^	0.23	1									
Fat	0.12	-0.08	-0.47^∗∗^	1								
Ash	-0.27	-0.25	0.10	-0.20	1							
CHO	0.04	-0.93^∗^	-0.27	0.03	-0.09	1						
TPC	0.29	0.27	-0.02	-0.02	-0.89^∗∗^	0.05	1					
Terpenoid	0.03	0.39^∗^	0.03	0.51^∗∗^	-0.74^∗∗^	-0.20	0.63^∗∗^	1				
Alkaloid	0.324^∗^	-0.35^∗^	0.24	-0.23	-0.09	0.38^∗^	0.11	-0.07	1			
TAA	-0.23	-0.39^∗^	0.14	-0.40^∗^	0.91^∗∗^	0.12	-0.84^∗∗^	-0.92^∗∗^	0.06	1		
Lycopene	-0.13	-0.38^∗^	-0.07	0.15	-0.57^∗∗^	0.60^∗∗^	0.62^∗∗^	0.44^∗∗^	0.19	-0.53^∗∗^	1	
Vitamin A	-0.19	0.31	0.10	0.11	-0.63^∗∗^	0.54^∗∗^	0.64^∗∗^	0.54^∗∗^	0.30	-0.57^∗∗^	0.94^∗∗^	1

^∗^ *N*: correlation is significant at 0.05 level; ^∗∗^*n*: correlation is significant at 0.01 level; CHO: carbohydrate; TPC: total phenolic contents; TAA: total antioxidant activities.

## Data Availability

All data are included in the manuscript.

## References

[1] Agume A., Njintang N., Mbofung C. (2017). Effect of soaking and roasting on the physicochemical and pasting properties of soybean flour. *Food*.

[2] Fernandes A. C., Nishida W., Da Costa Proença R. P. (2010). Influence of soaking on the nutritional quality of common beans (Phaseolus vulgaris L.) cooked with or without the soaking water: a review. *International Journal of Food Science and Technology*.

[3] Koffi-Nevry R., Koussémon M., Alloue-Boraud M., Kouassi K. (2013). Assessing the Microbiological level and the incidence of water-soaking on the proximate composition of two cultivars of cowpea (Vigna unguiculata L.) grains grown in Côte d’Ivoire. *British Microbiology Research Journal*.

[4] Siqueira B. d. S., Soares Júnior M. S., Fernandes K. F., Caliari M., Damiani C. (2013). Effect of soaking on the nutritional quality of pequi (Caryocar brasiliense Camb.) peel flour. *Food Science and Technology*.

[5] Maseta E., Mosha T. C., Nyaruhucha C., Laswai H. (2017). Nutritional quality of quality protein maize-based supplementary foods. *Nutrition & Food Science*.

[6] Mbela D. E. N., Kinabo J., Mwanri A. W., Ekesa B. (2018). Modification of local diets to improve vitamin A, iron and protein contents for children aged 6 to 23 months in Kagera, Tanzania. *African Journal of Food, Agriculture, Nutrition and Development*.

[7] Bentley J. W., Olanrewaju A. S., Madu T. (2017). *Cassava farmers' preferences for varieties and seed dissemination system in Nigeria: gender and regional perspectives*.

[8] FAO, IFAD, UNICEF, WFP, WHO The state of food security and nutrition in the world. Building climate resilience for food security and nutrition. 2018. http://www.fao.org/publications.

[9] Onyemachi V. C., Onyemachi S. E., Kalu C. E., Nnam R. E., Chigbo D. C. (2018). The effect of maturity on the proximate of orange fleshed sweet potato. *Annals Food Science and Technology*.

[10] Sayyad R. (2017). Effects of deep-fat frying process on the oil quality during French fries preparation. *Journal of Food Science and Technology*.

[11] Neela S., Fanta S. W. (2019). Review on nutritional composition of orange-fleshed sweet potato and its role in management of vitamin A deficiency. *Food Science & Nutrition*.

[12] Low J. W., Mwanga R. O. M., Andrade M., Carey E., Ball A. M. (2017). Tackling vitamin A deficiency with biofortified sweetpotato in sub-Saharan Africa. *Global Food Security*.

[13] Food and Agriculture Organization of the United Nations (FAO)/FAOSTAT (2019). *Statistical database, statistical Division Rome*.

[14] Wakil S. M., Ola J. O. (2018). Development of maize-tigernut fortified weaning food using starter cultures. *Food and Nutrition Sciences*.

[15] Olaniran A. F., Abiose S. H., Saka G. O. (2019). Nutritional quality and acceptability evaluation of Ogi flour biofortified with garlic and ginger. *Journal of Health Sciences*.

[16] Fikiru O., Bultosa G., Fikreyesus Forsido S., Temesgen M. (2017). Nutritional quality and sensory acceptability of complementary food blended from maize (Zea mays), roasted pea (Pisum sativum), and malted barley (Hordium vulgare). *Food Science & Nutrition*.

[17] Allen L., deBenoist B., Dary O., Hurrell R. (2006). *Guidelines on Food Fortification with Micronutrients*.

[18] Olaniran A. F., Okonkwo C. E., Owolabi A. O., Osemwegie O. O., Badejo T. E. (2020). Proximate composition and physicochemical properties of formulated cassava, cowpea and potato flour blends. *IOP Conference Series: Earth and Environmental Science*.

[19] Awolu O. O., Oseyemi G. F. (2016). Physicochemical and rheological properties of optimised cocoyam-based composite flour comprising cassava starch. *Acta Universitatis Cibiniensis. Series E: Food Technology*.

[20] Wireko-Manu F. D., Otu B. M., Aryeetey E., Quarcoo C., Oduro I. (2016). Acceptability of instant cassava-soybean based complementary food by weaning mothers. *British Journal of Applied Science & Technology*.

[21] Oyarekua M. A. (2009). Co-fermentation of cassava/cowpea/carrot to produce infant complementary food of improved nutritive quality. *Asian Journal of Clinical Nutrition*.

[22] Maseta E., Mosha T. C. E., Nyaruhucha C. N., Laswai H. (2016). Sensory evaluation of extruded quality protein maize-based supplementary foods. *African Journal of Food Science*.

[23] Rodríguez-Sandoval E., Fernández-Quintero A., Sandoval-Aldana A., Cuvelier G. (2008). Effect of processing conditions on the texture of reconstituted cassava dough. *Brazilian Journal of Chemical Engineering*.

[24] Alawode E. K., Idowu M. A., Adeola A. A., Oke E. K., Omoniyi S. A. (2017). Some quality attributes of complementary food produced from flour blends of orange flesh sweetpotato, sorghum, and soybean. *Croatian Journal of Food Science and Technology*.

[25] AOAC (2010). Official methods of analysis of AOAC International Gaithersburg MD. https://www.worldcat.org/title/official-methods-of-analysis-of-aoac-international/oclc/649275444.

[26] Yusuf A. J., Abdullahi M. I. (2019). The phytochemical and pharmacological actions of Entada africana Guill. & Perr.. *Heliyon*.

[27] Uddin M. S., Hossain M. S., Al Mamun A. (2018). Phytochemical analysis and antioxidant profile of methanolic extract of seed, pulp and peel of Baccaurea ramiflora Lour. *Asian Pacific Journal of Tropical Medicine*.

[28] Olaniran A. F., Abiose S. H. (2019). Nutritional evaluation of enhanced unsieved Ogi paste with garlic and ginger. *Preventive Nutrition and Food Science*.

[29] Olaniran A. F., Abiose S. H. (2018). Proximate and antioxidant activities of bio-preserved Ogi flour with garlic and ginger. *F1000Research*.

[30] Olaniran A., Abiose S. H., Gbadamosi S. O. (2013). Effect of ginger and garlic as biopreservatives on proximate composition and antioxidant activity of tomato paste. *Ife Journal of Technology*.

[31] Adebayo S. F. (2014). Effect of soaking time on the proximate, mineral compositions and anti-nutritional factors of lima bean. *Food Science and Quality Management*.

[32] Okudu H., Ojinnaka M. (2017). Effect of soaking time on the nutrient and antinutrient composition of bambara groundnut seeds (Vigna subterranean). *African Journal of Food Science and Technology*.

[33] Desalegn B. B. (2015). Effect of soaking and germination on proximate composition , mineral bioavailability and functional properties of chickpea flour. *Food Public Health*.

[34] Laryea D., Wireko-Manu F. D., Oduro I. (2018). Formulation and characterization of sweetpotato-based complementary food. *Cogent Food & Agriculture*.

[35] Ocheme O. B., Chinma C. E. (2008). Effects of soaking and germination on some physicochemical properties of millet flour for porridge production. *Journal of Food Technology*.

[36] Ojokoh A. O., Daramola M. K., Oluoti O. J. (2013). Effect of fermentation on nutrient and anti-nutrient composition of breadfruit (Treculia africana) and cowpea (Vigna unguiculata) blend flours. *African Journal of Agricultural Research*.

[37] Desalegn B. B., Abegaz K., Kinfe E. (2015). Effect of blending ratio and processing technique on physicochemical composition, functional properties and sensory acceptability of quality protein maize (QPM) based complementary food. *International Journal of Food Science and Nutrition Engineering*.

[38] Moghaddam S. M., Brick M. A., Echeverria D. (2018). Genetic architecture of dietary fiber and oligosaccharide content in a middle American panel of edible dry bean. *The Plant Genome*.

[39] World Health Organization (WHO) (2009). Infant Nutrition. *Infant and young child feeding*.

[40] Pan American Health (PAHO)/ World Health Organization (WHO) (2001). *Guiding principles for complementary feeding of the breastfed child*.

[41] Adenuga C. (2010). Nutritional and sensory profiles of sweet potato based infant weaning food fortified with cowpea and peanut. *Journal of Food Technology*.

[42] Amagloh F. K., Weber J. L., Brough L., Hardacre A., Anthony N., Coad J. (2012). Complementary food blends and malnutrition among infants in Ghana : a review and a proposed solution. *Scientific Research and Essays*.

[43] Pobee R. A., Akonor P. T., Bonsi E. (2017). Orange-fleshed sweet potato based complementary food provides sufficient vitamin A for infants aged 6-12 months. *African Journal of Food Science*.

[44] Odunmbaku L. A., Sobowale S. S., Adenekan M. K., Oloyede T., Adebiyi J. A., Adebo O. A. (2018). Influence of steeping duration, drying temperature, and duration on the chemical composition of sorghum starch. *Food Science & Nutrition*.

[45] Nestares T., Barrionuevo M., López-Frías M., Vidal C., Urbano G. (2003). Effect of different soaking solutions on nutritive utilization of minerals (calcium, phosphorus, and magnesium) from cooked beans (Phaseolus vulgaris l.) in growing rats. *Journal of Agricultural and Food Chemistry*.

[46] Vasishtha H., Srivastava R. P. (2013). Effect of soaking and cooking on dietary fibre components of different type of chickpea genotypes. *Journal of Food Science and Technology*.

[47] Felix I. N., Sheily N. E., Nkechinyere O. N., Sarah N. O. (2017). Effect of processing methods on the nutritional values and anti-nutritive factors of Adenanthera pavonina L. (Fabaceae) seeds. *African Journal of Biotechnology*.

[48] Akinyele I. O., Akinlosotu A. (1991). Effect of soaking, dehulling and fermentation on the oligosaccharides and nutrient content of cowpeas (Vigna unguiculata). *Food Chemistry*.

[49] Afify A. M. R., El-Beltagi H. S., El-Salam S. M., Omran A. A. (2012). Effect of soaking, cooking, germination and fermentation processing on proximate analysis and mineral content of three white sorghum varieties (Sorghum bicolor L. moench). *Notulae Botanicae Horti Agrobotanici Cluj-Napoca*.

[50] Nkhata S. G., Ayua E., Kamau E. H., Shingiro J. B. (2018). Fermentation and germination improve nutritional value of cereals and legumes through activation of endogenous enzymes. *Food Science & Nutrition*.

[51] Coffigniez F., Briffaz A., Mestres C., Alter P., Durand N., Bohuon P. (2018). Multi-response modeling of reaction-diffusion to explain alpha-galactoside behavior during the soaking-cooking process in cowpea. *Food Chemistry*.

[52] Boateng L., Nortey E., Ohemeng A. N., Asante M., Steiner-Asiedu M. (2019). Sensory attributes and acceptability of complementary foods fortified with Moringa oleifera leaf powder. *Nutrition & Food Science*.

[53] FOS C., Lopes K. H. (2016). Antioxidant potential of flours from cereals, tubers, beans and seeds chemical profile of Curcuma longa flour. *Journal of Nutrition & Food Sciences*.

[54] Abeshu Y., Kefale B. (2017). Effect of some traditional processing methods on nutritional composition and alkaloid content of lupin bean. *International Journal of Bioorganic Chemistry*.

[55] Olaniran A. F., Abiose S. H., Adeniran A. H. (2015). Biopreservative effect of ginger (Zingiber officinale) and garlic powder (Allium sativum) on tomato paste. *Journal of Food Safety*.

[56] Abdel-Rahman H. G., Abdelrazek H. M. A., Zeidan D. W., Mohamed R. M., Abdelazim A. M. (2018). Lycopene: hepatoprotective and antioxidant effects toward bisphenol A-induced toxicity in female Wistar rats. *Oxidative Medicine and Cellular Longevity*.

